# Effect of D-Ala-Ended Peptidoglycan Precursors on the Immune Regulation of *Lactobacillus plantarum* Strains

**DOI:** 10.3389/fimmu.2021.825825

**Published:** 2022-01-19

**Authors:** Xin Song, Fengjiao Li, Mingshu Zhang, Yongjun Xia, Lianzhong Ai, Guangqiang Wang

**Affiliations:** Shanghai Engineering Research Center of Food Microbiology, School of Health Science and Engineering, University of Shanghai for Science and Technology, Shanghai, China

**Keywords:** Ddl ligase, *Lactobacillus plantarum*, d-alanine, vancomycin, immune pathways

## Abstract

The resistance of *Lactobacillus plantarum* to vancomycin depends on its peptidoglycan composition. Vancomycin has poor binding affinity with peptidoglycan precursors ending in D-alanyl-D-lactate (D-Ala-D-Lac) but binds strongly to peptidoglycan precursors ending in D-alanyl-D-alanine (D-Ala-D-Ala), resulting in resistance and sensitivity, respectively. The ligase Ddl, which generates D-Ala-D-Lac or D-Ala-D-Ala incorporated into the peptidoglycan precursor chain, is responsible for this specificity. To study the effect of peptidoglycan precursors on immunity, we constructed several strains of *L. plantarum* expressing the *ddl* gene of *Lactococcus lactis* to change their peptidoglycan precursors. The change in the termini of the peptidoglycan precursors was determined by the sensitivity of the strains to vancomycin. The overexpression of *ddl* increased the susceptibility of the strains to vancomycin. We further explored the regulation of the macrophage inflammatory response pathway by the wild-type and constructed strains, and found that these strains induced the MyD88-dependent TRAF6/MAPK pathway, and the increase in D-Ala *L. plantarum* peptidoglycan precursors increased the secretion of the inflammatory factors IL-6, IL-1β and TNF-α. These results indicate that D-Ala-ended peptidoglycan precursors play a central role in the variable immunomodulatory ability of *L. plantarum*.

## Introduction

The cytoplasmic membrane in gram-positive bacteria is covered by a thick cell wall consisting of multiple layers of peptidoglycan, a polysaccharide composed of two alternating glucose derivatives, N-acetylglucosamine (NAG) and N-acetylmuramic acid (NAM). The chains are cross-linked to one another by a tetrapeptide that extends from the NAM sugar unit, allowing a lattice-like structure to form. The four amino acids that compose the tetrapeptide are L-alanine, D-glutamine, L-lysine or meso-diaminopimelic acid (DPA), and D-alanine (D-Ala) (1).

In peptidoglycan synthesis, the precursor pentapeptide Lipid II is generated with a terminal D-alanyl-D-alanine (D-Ala-D-Ala) or D-alanyl-D-lactate (D-Ala-D-Lac). This is the starting unit from which bacteria extend the sugar chain and crosslink the peptide chain outside the cell membrane to form a mature peptidoglycan. Under the action of transglycosylase, the Lipid II is linked to the sugar chain outside the cell membrane to make it extend horizontally. After the action of transpeptidase, peptide bonds are formed between the peptide tails of adjacent peptidoglycan monomers and terminal amino acid (D-alanine or D-lactate) is released, which leads to longitudinal cross-linking. Vancomycin combines with D-Ala-D-Ala-ended peptidoglycan precursors on the bacterial cell wall to form a stable non-covalent complex, thereby inhibiting the extension or/and cross-linking of peptidoglycan to repress cell wall synthesis of and eventually cause bacterial cell death (2, 3). *Lactobacillus plantarum* produces peptidoglycan precursors ending in D-lactate (D-Lac) instead of D-Ala, leading to intrinsic resistance to vancomycin. *L. plantarum* expresses the D-Ala-D-Lac ligase (Ddl_Lp_), which plays a central role in this specificity by synthesizing D-Ala-D-Lac depsipeptides that are added to the precursor peptide chain (4), and D-Lac dehydrogenase for D-Lac production, which affects peptidoglycan biosynthesis (5). In contrast, *Lactobacillus lactis* exclusively produces D-Ala-ended peptidoglycan precursors through D-Ala-D-Ala ligase (Ddl_Lc_) action, resulting in natural sensitivity to high levels of vancomycin (6).

*L. planta*rum can produce D-Lac or L-lactate (L-Lac) through the activity of D-Lac or L-Lac dehydrogenase, respectively (7). A mutant strain of *L. plantarum* lacking both D- and L-Lac dehydrogenase activities was reported to produce only trace amounts of D- and L-Lac, thereby seriously affecting the peptidoglycan synthesis pathway (2). Although the wild-type precursor was still present, the mutant strain substantially synthesized novel D-Ala-ended precursors and showed a highly enhanced sensitivity to vancomycin (2). D-Ala on the teichoic acid (TA) branch chain caused corresponding immunological responses and triggered the immune response pathway. Purification of lipoteichoic acid (LTA) from *Staphylococcus aureus* caused the loss of alanine substituents and weaker cytokine induction activity than that observed with unpurified LTA. The hydrolysis of active LTA alanine substituents also greatly reduced the induction of cytokines (8). In *in-vivo* experiments, the *L. plantarum* mutant was found to incorporate much less D-Ala in TAs than the wild-type strain. This deficiency significantly impacted bacteria-induced immunomodulatory reactions, with a significant decrease in proinflammatory cytokines secreted by peripheral blood mononuclear cells and monocytes stimulated by the mutant strain compared with the parental strain (9).

Toll-like receptors (TLRs) are the main pattern recognition receptors on the host cells that recognize microorganism, inducing a broad spectrum of extracellular and intracellular signaling pathways. Nuclear factors-kappa β (NF-κβ) is a downstream hub of TLR signaling pathways (10), and is present in almost all cells. When a cell is stimulated, pathogen-associated molecular patterns, in combination with TLRs, send a signal to intracellular receptor. The Toll/interleukin (IL)-1 receptors (TIR) region of TLRs binds to the carboxyl terminal of MyD88, which then recruits interleukin-1 receptor associated kinases (IRAK) and induces phosphorylation of IRAK1. IRAK1 interacts with TNF6 to activate the IKK complex and MAPK. The IKK complex phosphorylates IκB kinase, which is then ubiquitinated and degraded by ubiquitin ligase (11). NF-κβ, which binds to IκB, is activated, and enters the nucleus to regulate the transcriptional expression of target genes (12), and then leads to inflammation, immunity, and various pathological reactions (13). INOS is a key enzyme in NO synthesis and induces NO production. The high expression of iNOS can produce excessive NO and reactive nitrogen and cause cell inflammatory damage.

Probiotics improving immunity (14, 15), enrich beneficial intestinal bacteria (16, 17) and inhibit the production of inflammatory factors related to intestinal diseases (18, 19). Certain probiotics can regulate the TLR NF-κβ signaling pathway and the expression of inflammatory factors, improving intestinal mucosal inflammation (20). Peptidoglycan, one of the most abundant microbe-associated molecular patterns (MAMPs) in lactic acid bacteria (LAB), activates the host’s antigen-presenting cells by binding to TLRs. This binding induces cell surface receptors expression to regulate cell functions, while stimulating the host cells to secrete cytokines and chemokines to regulate the immune response (21).

Peptidoglycan, wall TA and LTA have immunomodulatory effects, and a change in the D-Ala content of *L. plantarum* LTA was shown to significantly affect the immunomodulatory responses induced by the bacterium (22). It is not yet known whether D-Ala derived from peptidoglycans has a similar immunomodulatory effect. In this work, we overexpressed the gene encoding *L. lactis* dipeptide ligase (*ddl*) into *L. plantarum* AR113 and Lac dehydrogenase double knockout (AR113_△ldhL△ldhD_) strains. To validate changes in the peptidoglycan precursor termini, we investigated the sensitivity of these strains to high levels of vancomycin. The constructed strains were evaluated *in-vitro* to explore the effects of D-Ala-ended peptidoglycan precursors on immune reaction. This study helps to identify the role of D-Ala in the immunomodulator ability of *L. plantarum*, and provides a theoretical basis for the screening and application of probiotics.

## Materials and Methods

### Bacterial Strains, Media, and Culture Conditions

*L. plantarum* AR113, *L. plantarum* AR113_△ldhL△ldhD_ and *L. lactis* NZ9000 were obtained from Shanghai Engineering Research Center of Food Microbiology, University of Shanghai for Science and Technology (Shanghai, China). *L. plantarum* AR113 and *L. plantarum* AR113_△ldhL△ldhD_ were streaked on separate de Man, Rogosa and Sharpe (MRS, Oxoid Ltd, Basingstoke, UK) agar plates, and incubated in an anaerobic workstation at 37°C for 24 h before experimental use. *L. plantarum* were grown at 37°C in MRS broth in the presence of 50 mM L-lactate (98%, Shanghai yuanye Bio-Technology Co., Ltd) or D-lactate (90%, Shanghai yuanye Bio-Technology Co., Ltd) when minimum inhibitory concentration (MIC) was measured. *L. lactis* NZ9000 was grown in M17 medium (Difco Laboratories, MI, U.S.A) supplemented with 0.5% glucose (GM17) at 30°C for 36 h before experimental use. *E. coli* top10 was used as a host for molecular cloning and was grown at 37°C in Luris-Bertani broth (LB, Gibco, CA, USA).

### DNA Manipulation and Cloning Procedure

*Ddl* was amplified by PCR using *L. lactis* NZ9000 chromosomal DNA as a template with the primers 5’- atatgaatgacaatga tgttggatccatgtcaaaagaa acT -3’ and 5’- cacgggaaaatcatctcttataactc gagatctatcgata agcttaag -3’. The PCR product was purified for sequencing by the BGI Company (Shenzhen, China). The correct sequence was cleaved by the restriction endonucleases BamHI and Xho I and subcloned into the pIB184 plasmid predigested at the same restriction sites. The resulting recombinant plasmid was named as pIB184-Ddl_Lc_.

### Transformation by Electroporation

*L. plantarum* competent cells were prepared as previously described (23). Electro transformation of *L. plantarum* was performed as follows. Briefly, a 3-5% (v/v) inoculum from an overnight culture was inoculated into 50 mL of SGMRS (50 mL MRS with 0.3 M sucrose and 1% glycine), and incubated at 37°C until the mid-exponential phase was achieved (OD_600_ of 0.3-0.5). The cells were recovered by centrifugation at 4500 rpm and 4°C for 10 min. The cells were washed twice with SM buffer (952 mM sucrose, 5 mM MgCl_2_), and resuspended in 800 μL of SM buffer. The competent cells were aliquoted and stored at −80°C. Subsequently, 100 μL of the cell suspension and a maximum of 1μL of the plasmid DNA solution were electroporated using an Eporator electroporator (Bio-Rad, UK) in cuvettes with a 2-mm electroporation gap at 2.5 kV, capacitance of 4 ms, and parallel resistance of 400 Ω.

After electroporation, the cells were immediately resuspended in 800 μL of SGMRS and kept at 37°C for 3 h for recovery. Then, 100 μL of the cells were spread on MRS agar plates containing 200 μg/mL erythromycin and incubated at 37°C for 36-48 h. Transformants grown on resistant plates were further screened by plasmid isolation and restriction enzyme analysis.

### Minimum Inhibitory Concentration of Vancomycin

The bacteria were streaked on separate MRS agar plates containing 200 μg/mL erythromycin (Sangon Biotech, Shanghai, China), and the plates were incubated in an anaerobic workstation at 37°C. After 2 days of culture, single colonies of bacteria were separately activated for two generations in MRS liquid medium containing 200 μg/mL erythromycin at 37°C for 16 h. Subsequently, a 3-5% (v/v) inoculum from an overnight culture was inoculated into 10 mL MRS liquid medium containing 0, 0.5, 2, 16, and 256 μg/mL of vancomycin.

### Cell Culture Experiments

RAW264.7 mouse macrophages were activated and maintained using Dulbecco’s modified Eagle’s medium (DMEM, Gibco™, Thermo Fisher Scientific, Grand Island, USA) containing 10% fetal bovine serum, 100 U/mL penicillin, and 100 μg/mL streptomycin at 37°C under a humidified atmosphere of 5% CO_2_.

### RNA Extraction and RT-PCR Gene Expression Analysis

RT-PCR was performed using a LightCycler^®^96 instrument (Roche China). cDNA was synthesized using a HiScript ^®^III RT SuperMix for qPCR (+gDNA wiper) from 500 ng of total RNA extracted from RAW264.7 cells. Then, qPCR was performed using a UNICON™qPCR SYBR^®^Green Master Mix (Yeasen Biotechnology, Shanghai, China) and β-actin was used as an internal reference gene. The procedure of quantitative PCR was as follows: 95°C for 30 s, 95°C for 5 s, 40 cycles, at optimum annealing temperature for 30 s. The results were analyzed by the method of 2^-ΔΔCt^. The primer sequences are shown in **Table 1**.

**Table 1:** RT-PCR primer sequence.

Primer	Sequences of the primer	Tm(°C)	Length(bp)
TNF-α	F: CCTGTAGCCCACGTCGTAG	58	148
	R: GGGAGTAGACAAGGTACAACCC	57	
IL-1β	F: CCCTGCAGCTGGAGAGTGTGG	63	153
	R: TGTGCTCTGCTTGAGAGGTGCT	61	
IL-6	F: ATGAACTCCTTCTCCACAAGCGC	61	628
	R: GAAGAGCCCTCAGGCTGGACTG	62	
iNOS	F: GCTCGCTTTGCCACGGACGA	63	146
	R: AAGGCAGCGGGCACATGCAA	65	
TGF-β	F: AAGACTTCACCCCAAAGCTGG	59	177
	R: TGAGCGCTCTCTGAGATCCAA	58	
Myd88	F: CTGGCCTTGTTAGACCGTGA	58	209
	R: TCGAAAAGTTCCGGCGTTTG	58	
IL-18R	F: TTTGCTTGACCGAGATGTGACC	58	524
	R: GCCTGATCCACACAGCAAGTTC	60	
Cox-2	F: GTTCATCCCTGACCCCCAAG	57	378
	R: ACTCTGTTGTGCTCCCGAAG	59	
ERK	F: TGACCTCAAGCCTTCCAACC	58	88
	R: ATCTGGATCTGCAACACGGG	59	
TRAF6	F: CACCACCATCAAGGACTCAA	56	102
	R: GAGACAGAGGCAACCTGACC	59	
P38	F: TCACGCCAAAAGGACCTACC	57	107
	R: ATTCCTCCAGTGACCTTGCG	59	
JNK	F: ATTGAACAGCTCGGAACACC	57	140
	R: GAGTCAGCTGGGAAAAGCAC	57	
β-actin	F: GGCTGTATTCCCCTCCATCG	57	154
	R: CCAGTTGGTAACAATGCCATGT	57	

### Statistical Analysis

The gene sequencing result were analyzed using Vector NTI (Thermo Fisher Scientific, Waltham, MA, U.S.A). GraphPad Prism 8 (GraphPad Software, San Diego, CA, USA) was used for the figures, and SPSS (IBM Corp., Armonk, NY, U.S.A) software was used for statistical analysis. Shapiro–Wilk test was used to verify the normality of data and Levene’s test was used to assess equal variance of data. Statistical comparison of two groups was performed using Student’s t-test, or Wilcoxon–Mann–Whitney test (when normality test fails). Multiple groups were compared with one-way ANOVA. The statistical analyses were assessed using one-way ANOVA. Data are presented as the means with standard deviations and differ significantly as presented with different letters at *P* < 0.05.

## Results

### Construction of Recombinant Strains and Sensitivity to Vancomycin

**Figure 1:**
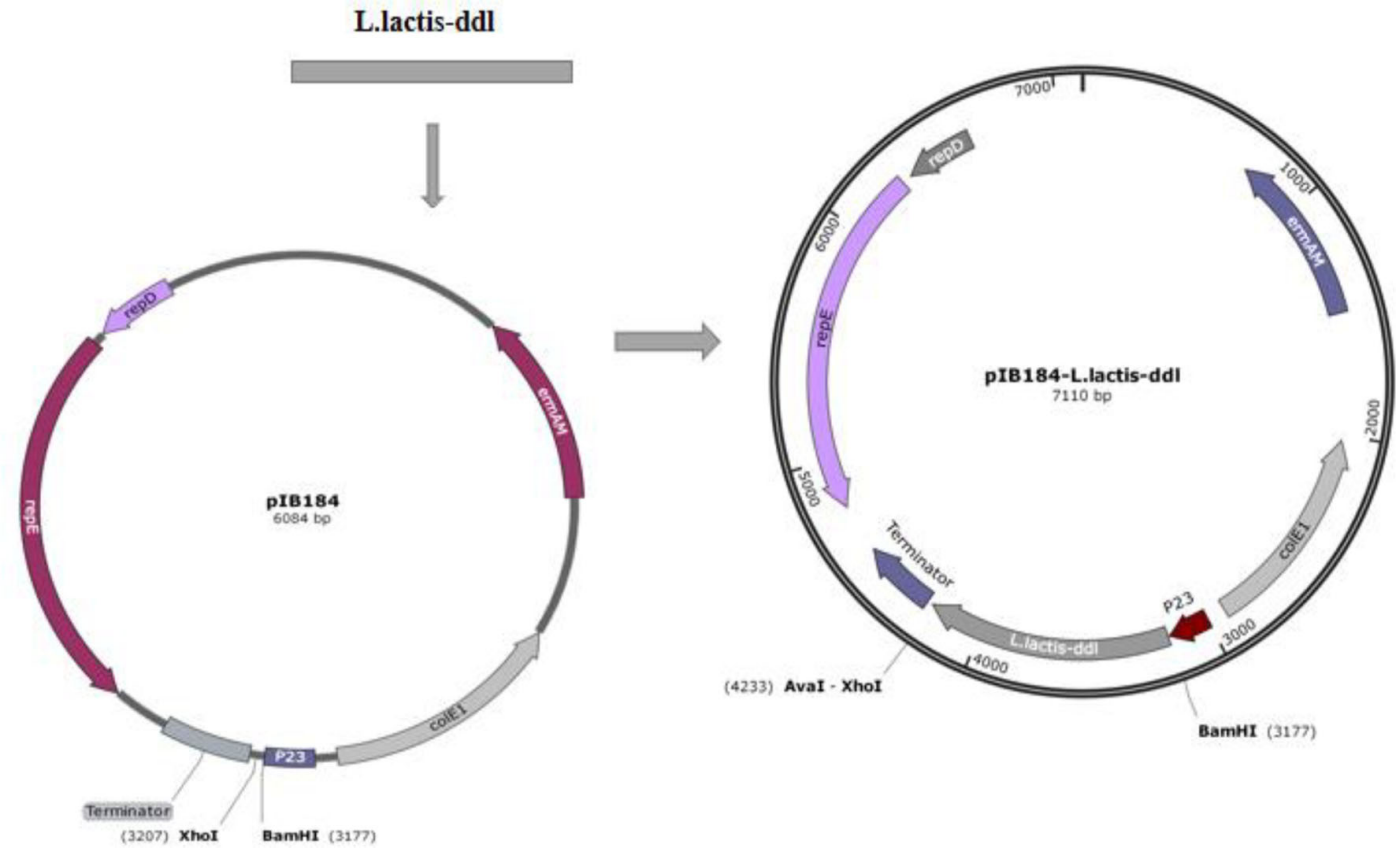
Construction of the recombinant plasmid pIB184-DdlLc.

*L. plantarum* AR113_△ldhL△ldhD_ is a mutant of *L. plantarum* AR113 in which the *ldhL* and *ldhD* genes are knocked out. These key genes regulate the production of L-Lac dehydrogenase and D-Lac dehydrogenase, which catalyze pyruvate to produce L-Lac and D-Lac respectively. The correct *ddl* fragment was digested by BamHI and XhoI, and ligated into a pIB184 vector, containing the constitutive promoter P23 from LAB to produce the recombinant plasmid pIB184-Ddl_Lc_. The recombinant plasmid was verified by PCR and double digestion. pIB184-Ddl_Lc_ was transformed into *L. plantarum* AR113 and *L. plantarum* AR113_△ldhL△ldhD_ competent cells by electroporation to yield the strains AR113-Ddl_Lc_ and AR113_△ldhL△ldhD_-Ddl_Lc_ (**Figure 1**). The *ldhL* and *ldhD* knockout strain we constructed barely produced lactic acid and consequently provided little D-Lac for peptidoglycan precursor production. Therefore, exogenous D-LAC was added to remedy this defect. We tested the sensitivity of the wild-type strain and Ddl overexpressing strains to vancomycin to observe any effects related to the change in peptidoglycan precursor termini. *L. plantarum* AR113_△ldhL△ldhD_ showed high sensitivity to vancomycin, which was mitigated by supplementation with exogenous D-Lac. Similar results were observed for *L. plantarum* AR113_△ldhL△ldhD_-Ddl_Lc_. These results indicated that *L. plantarum* AR113_△ldhL△ldhD_ and AR113_△ldhL△ldhD_-Ddl_Lc_ produced peptidoglycan precursors ending in D-Lac, like those produced by the wild-type AR113 strain, after supplementation with exogenous D-Lac, thus reducing sensitivity to vancomycin (**Table 2**).

**Table 2:** MICs of vancomycin in wild-type and mutant strains of *L. plantarum* (μg/mL).

*L. plantarum* strain	MRS	MRS+50 mM L-lactate	MRS+50 mM D-lactate
AR113	>256	>256	>256
AR113_ΔldhL△ldhD_	2	2	>256
AR113_ΔldhL△ldhD_-Ddl_Lc_	0.5	0.5	>256

### Analysis of the Immunomodulatory Signaling Pathway Induced by Ddl-Overexpressing Strains

**Figure 2:**
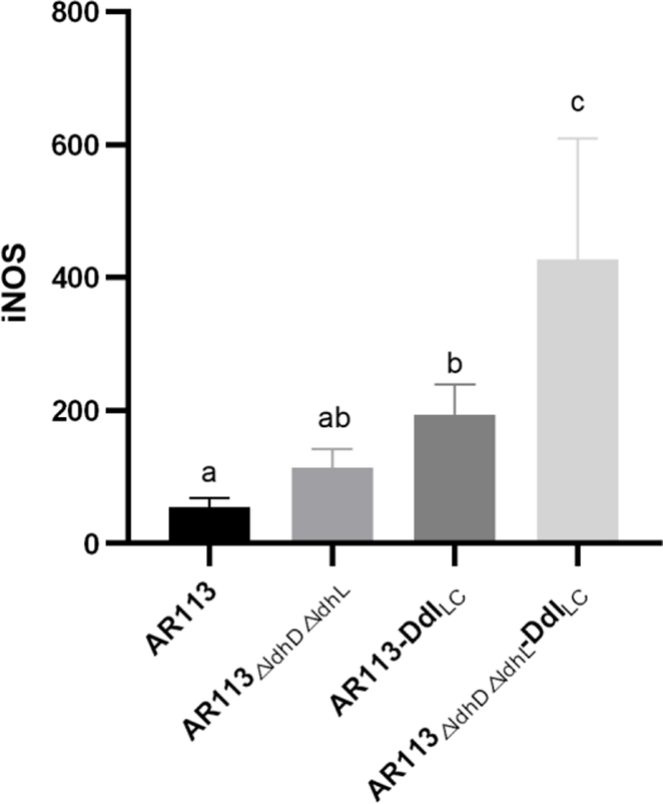
Effect of Ddl-overexpressing *L. plantarum* strains on iNOS production in macrophages. The statistical analyses were assessed using one-way ANOVA. Data are represented as means ± standard deviations (n = 7). Data indicated with different letters differ significantly (P < 0.05).

Excessive secretion of inducible nitric oxide synthase (iNOS) can induce the production of large amounts of nitric oxide (NO), resulting in inflammatory cell damage. Compared with the wild-type *L. plantarum* AR113 strain, the three strains engineered to overexpress Ddl led to greater increases in the expression of iNOS mRNA. The increase in D-Ala-ended peptidoglycan on the bacterial cell wall increased the expression of the gene encoding iNOS (**Figure 2**).

**Figure 3:**
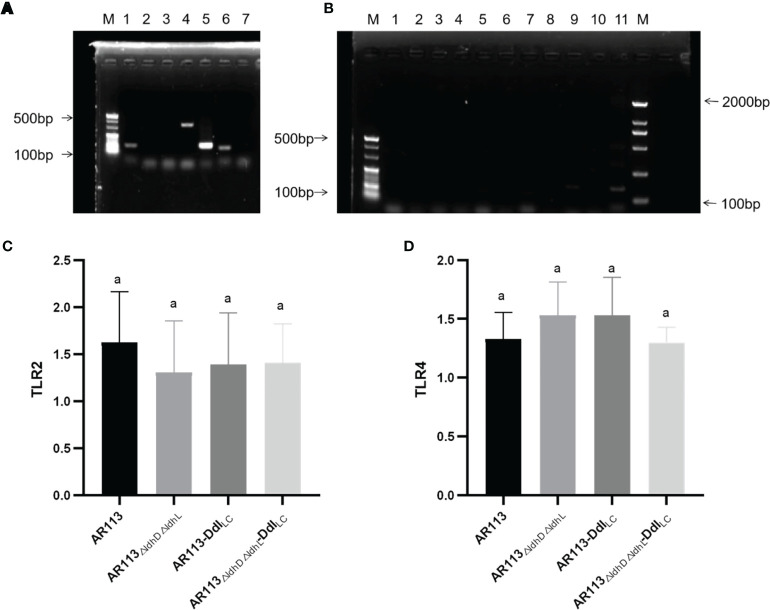
Analysis of immunomodulatory signaling pathways affected by Ddl-overexpressing strains. **(A)** lane 1: p38, lane2: JNK, lane 3: IRAK1, lane 4: p38MAPK, lane 5: TRAF6, lane6: ERK; **(B)** lane 1: TLR1, lane 2: TLR3, lane 3: TLR5, lane 4: TLR6, lane 5: TLR7, lane 6: TLR8, lane 7: TLR9, lane 8: Iκκ-β, lane 9: Iκκ-γ, lane 10: TRIF, lane 11: NF-κβP56; **(C)** RT-PCR of TLR2; **(D)** RT-PCR of TLR4. The statistical analyses were assessed using one-way ANOVA. Data are represented as means ± standard deviations (n = 7). Data indicated with different letters differ significantly (P < 0.05).

*L. plantarum* cell wall peptidoglycan has an immune stimulatory effect and thus can bind to TLR2 and other receptors on macrophages, activate cell signal transduction, and induce downstream related to immune. Using PCR, the ability of D-Ala to induce the TLR2, TLR4 and MAPK signal transduction pathways was verified, whereas activity through the TRIF and Iκκ pathways was not verified (**Figure 3A**). This indicated that the strains bound to TLR2 and TLR4 on the surfaces of macrophages and entered the cells, and then triggered MyD88-dependent pathways. The ability of *L. plantarum* AR113, AR113_△ldhL△ldhD_ and Ddl overexpressing strains binding to TLR2 and TLR4 was not significantly different (**Figures 3B–D**).

**Figure 4:**
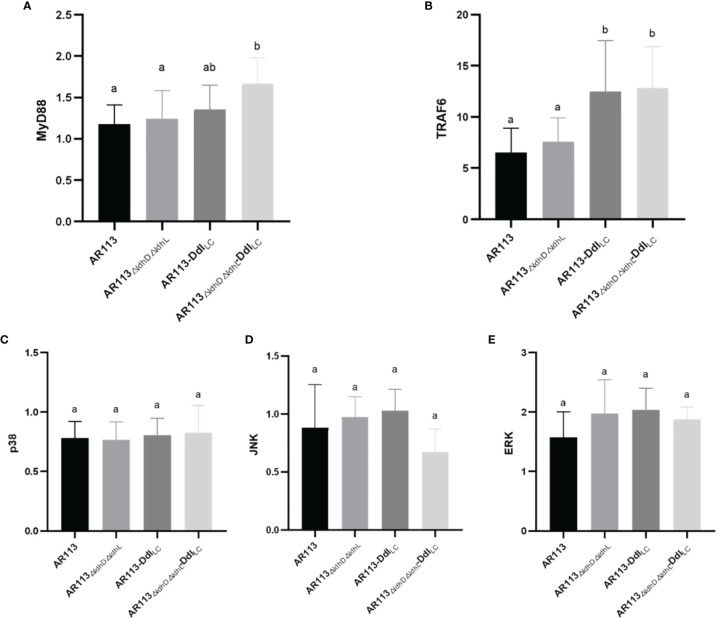
Analysis of the immunomodulatory signaling pathway affected by Ddl-overexpressed strains. **(A)** MyD88; **(B)** TRAF6; **(C)** p38; **(D)** JNK; **(E)** ERK. The statistical analyses were assessed using one-way ANOVA. Data are represented as means ± standard deviations (n = 7). Data indicated with different letters differ significantly (P < 0.05).

The wild-type and Ddl overexpressing strains were co-cultured with macrophages, and the expression of transcription factors was measured in the latter cells. MyD88 plays a crucial role in signal transduction pathways involving IL-1 and TLRs. RT-PCR analysis showed that *L. plantarum* AR113_△ldhL△ldhD_-Ddl_Lc_ intensely induced MyD88 mRNA expression, whereas *L. plantarum* AR113 and AR113-Ddl_Lc_ induced weaker MyD88 mRNA expression. Ddl-overexpressing strains increased the expression of MyD88 mRNA in macrophages downstream of the TLR2 pathway (P < 0.05). Compared with *L. plantarum* AR113, AR113_ΔldhL△ldhD_ did not significantly increase the expression of MyD88 mRNA (**Figure 4A**). TRAF6 plays a key role in innate and adaptive immune responses. The expression of TRAF6 mRNA was stimulated by Ddl-overexpressing AR113 strains (P < 0.05), and the strain lacking *ldhL* and *ldhD* induced activation of TRAF6 to a greater degree than MyD88 (**Figure 4B**). The activation of ERK, p38, and JNK in MAPK pathways were observed. All four *L. plantarum* strains induced comparable levels of p38, ERK, and JNK phosphorylation, suggesting that these strains can induce MyD88-dependent TRAF6/MAPK pathway signal transductions (**Figures 4C–E**).

### Effects of D-Ala-ended *L. plantarum* Peptidoglycan Precursors on Cytokine Production

**Figure 5:**
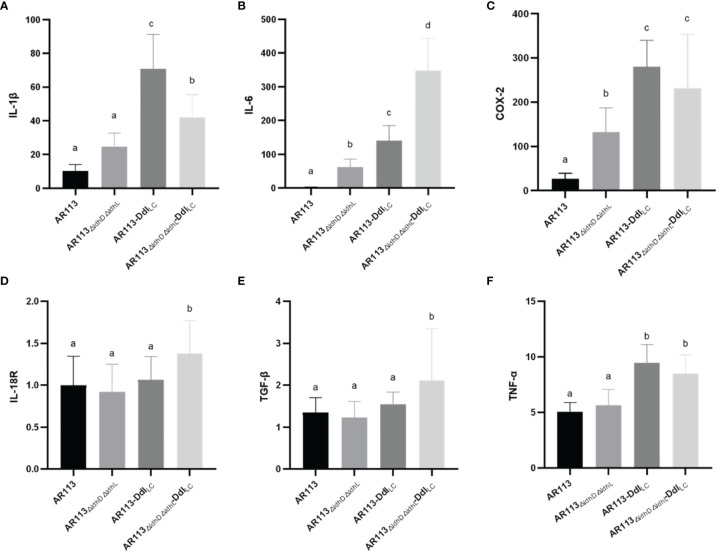
Effects of D-Ala-ended peptidoglycan precursors on the expression of cytokine/inflammatory factor genes **(A)** IL-1β, **(B)** IL-6, **(C)** COX-2, **(D)** IL-18R, **(E)** TGF-β and **(F)** TNF-α in macrophages. The statistical analyses were assessed using one-way ANOVA. Data are represented as means ± standard deviations (n = 7). Data indicated with different letters differ significantly (P < 0.05).

To investigate the functional role of D-Ala at the termini of the of *L. plantarum* peptidoglycan precursors in the production of cytokine and expression of other factors, RAW264.7 macrophages were stimulated with *L. plantarum* AR113, AR113*-*Ddl_Lc_, AR113_△ldhL△ldhD_, and AR113_△ldhL△ldhD_-Ddl_Lc_. As shown in **Figure 5**, all four strains induced the expression of mRNAs encoding the cytokines IL-6, IL-1β, COX-2, IL-18 R, TGF-β, TNF-α, although relatively lower levels of gene expression were induced by the AR113 and AR113_△ldhL△ldhD_ strains. These results indicate that D-Ala can induce the expression of genes encoding cytokines IL-6, IL-1β, COX-2 (**Figure 5**).

## Discussion

Ddl synthesizes D-Alanyl-D-lactate or D-alanyl-D-alanine, which are added to the precursor peptide chain by the enzyme MurF and contribute to the sensitivity of *L. lactis* to vancomycin. An active site phenylalanine or tyrosine in the Ddl determines depsipeptide or dipeptide activity, which results in resistance and sensitivity to vancomycin, respectively. Ddl from *L. plantarum* encodes a depsipeptide ligase which synthesizing D-Ala-D-Lac depsipeptides, while *L. lactis* expresses the D-Ala-D-Ala ligase (Ddl_Lp_). In addition, the resistance of these strains to vancomycin was related to the amount of D-Ala at the termini of the peptidoglycan precursors. The loss of D-Lac dehydrogenase, the key enzyme in the formation of lactate from pyruvate, reduced D-Lac production and affected the sensitivity of the strain to vancomycin. Our study found Ddl_Lc_ was overexpressed in *L. plantarum* to induce D-Ala-D-Ala dipeptide production and render the strain sensitive to vancomycin. When the strain was provided with exogenous D-Lac, the results were reversed, indicating that Ddl_Lp_ promoted the production of large amounts of D-Ala-D-Lac for the synthesis of D-Lac-ended peptidoglycan precursors. Although D-Lac is also produced by the racemization of L-Lac in *L. plantarum* (24), the tested strains still showed vancomycin sensitivity when supplemented with exogenous L-Lac. This shows that only a certain amount of D-lac can change the sensitive to vancomycin. This also indicates that we have indeed changed the structure of the peptidoglycan precursor by knocking out the gene of Lac dehydrogenase or overexpressing the gene of Ddl_Lc_.

Large molecular substances in the *L. plantarum* cell wall and cell membrane surface induce a series of immune responses by binding to TLRs on the surfaces of macrophages and entering the cells, where they can trigger both MYD88-dependent and independent pathways. In our study, we co-cultured *L. plantarum* AR113, AR113-Ddl_Lc_, AR113_△ldhL△ldhD_, AR113_△ldhL△ldhD_-Ddl_Lc_ with RAW264.7 macrophage. Endogenous NO, a small molecule with a wide range of complex biological activities, is involved in a variety of pathophysiological processes and exerts certain cytotoxic side effects at high concentrations. iNOS is a key enzyme in the synthesis of NO (25), and thus associated with inflammatory cell damage (26). *L. plantarum* AR113_△ldhL△ldhD_, which theoretically produced the highest level of D-Ala-ended peptidoglycan precursors among the tested strains, significantly promoted the expression of iNOS mRNA, which was measured at levels more than four times higher than those induced by the wild-type *L. plantarum* AR113 strain. We further confirm the expression of pathway-related factors in the macrophages by PCR. Only TLR2 and TLR4 had DNA bands. This result indicated that these four strains (AR113, AR113-Ddl_Lc_, AR113_△ldhL△ldhD_ and AR113_△ldhL△ldhD_-Ddl_Lc_) induced TLR2 and TLR4 pathway activity and thus induced the TRAF6/MAPK pathway but not TRIF or Iκκ pathways. This is exactly in line with the immune response caused by *Lactobacillus plantarum* AR113 (27). The overexpression of Ddl_LC_ increased the level of D-Ala at the peptidoglycan termini of potently induced the production of proinflammatory cytokines by macrophages in co-culture. As previously reported, the loss of D-Ala on LTA branch chains of *L. plantarum* resulted in certain anti-inflammatory effects, indicating that D-Ala itself has pro-inflammatory effects (6). Therefore, the content of D-ala in the cell wall may be used to characterize the immunomodulatory ability of the strain. Ddl or Lac dehydrogenase can determine the change of D-ala content in the strain. Therefore, they may be used to quickly screen for immunomodulatory strains. Overall, our research provides a potential theoretical basis for the application or screening of probiotics.

## Data Availability Statement

The original contributions presented in the study are included in the article/supplementary material. Further inquiries can be directed to the corresponding author.

## Author Contributions

Methodology: XS, FL, and MZ. Data curation: FL, MZ, and GW. Investigation: XS, FL, YX, and MZ. Resources: YX, and LA. Writing—Original draft: XS, FL, MZ, and GW. Editing: LA. Writing-Review and Editing: XS, LA, and GW. All authors contributed to the article and approved the submitted version.

## Funding

This study was funded by Shanghai Agriculture Applied Technology Development Program, China (grant 2019-02-08-00-07-F01152), National Science Foundation for Distinguished Young Scholars of China (No. 32025029).

## Conflict of Interest

The authors declare that the research was conducted in the absence of any commercial or financial relationships that could be construed as a potential conflict of interest.

## Publisher’s Note

All claims expressed in this article are solely those of the authors and do not necessarily represent those of their affiliated organizations, or those of the publisher, the editors and the reviewers. Any product that may be evaluated in this article, or claim that may be made by its manufacturer, is not guaranteed or endorsed by the publisher.
